# Supplementary figures and images for: Tuber melanosporum shapes nirS-type denitrifying and ammonia-oxidizing bacterial communities in Carya illinoinensis ectomycorrhizosphere soils
Source: PeerJ. 2020 Sep 3;8:e9457. doi: 10.7717/peerj.9457 (PMC7474878; doi:10.7717/peerj.9457)

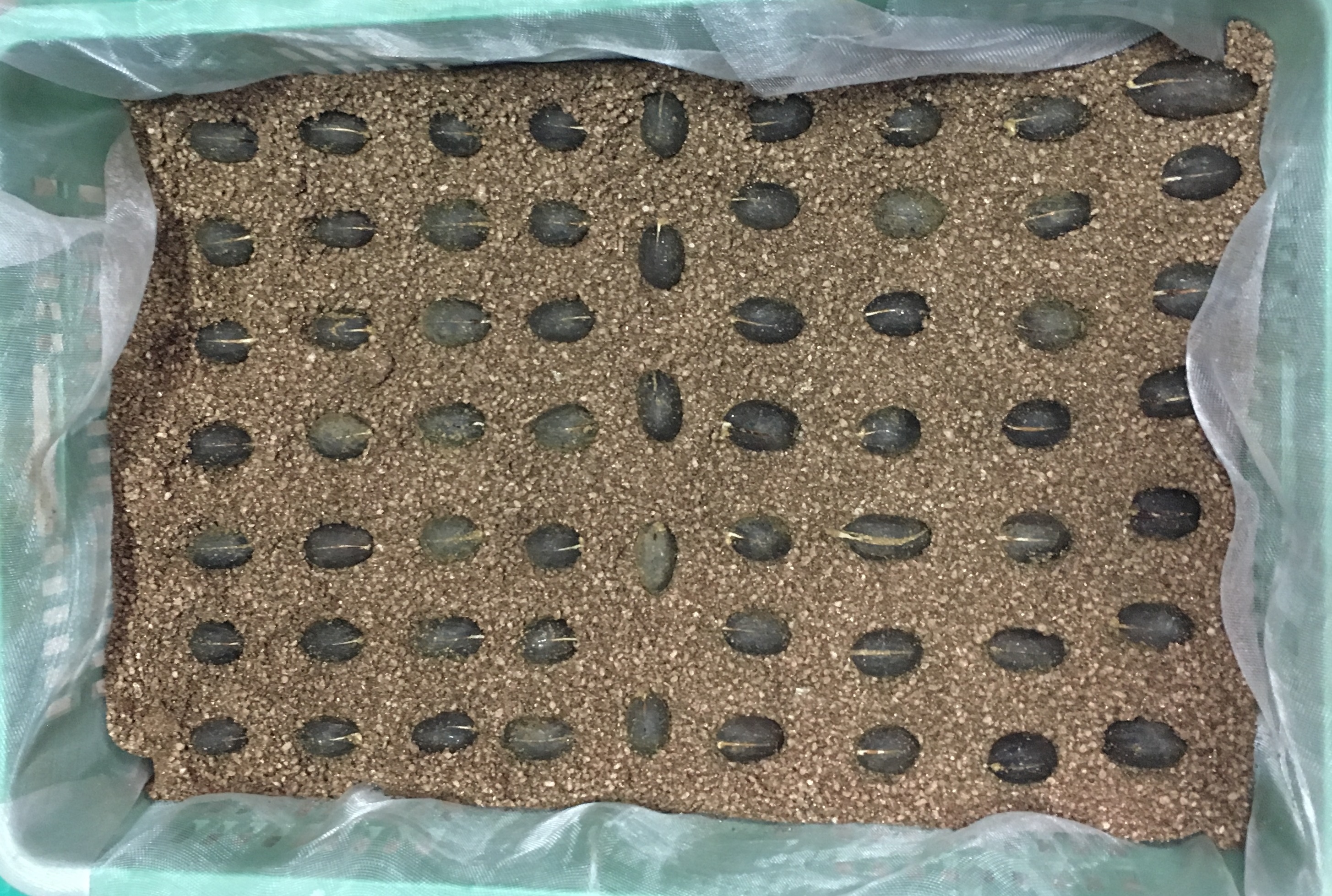

Supplement: Figure S1 — Seeds were sown in a prepared matrix, and sprinkled with disinfected substrate (vermiculite, perlite organic soil and water at a ratio of 1:1:1:0.9, v/v/v/v) [file peerj-08-9457-s001.jpg]
