# Supplementary material for: Tuber melanosporum shapes nirS-type denitrifying and ammonia-oxidizing bacterial communities in Carya illinoinensis ectomycorrhizosphere soils
Source: PeerJ. 2020 Sep 3;8:e9457. doi: 10.7717/peerj.9457 (PMC7474878; doi:10.7717/peerj.9457)
Supplement: Figure S2 — FH, ectomycorrhizosphere soil (the rhizosphere soil of Carya illinoinensis mycorrhized with Tuber melanosporum). CK, rhizosphere soil from cultivated Carya illinoinensis without Tuber melanosporum partner. [file peerj-08-9457-s002.pdf]

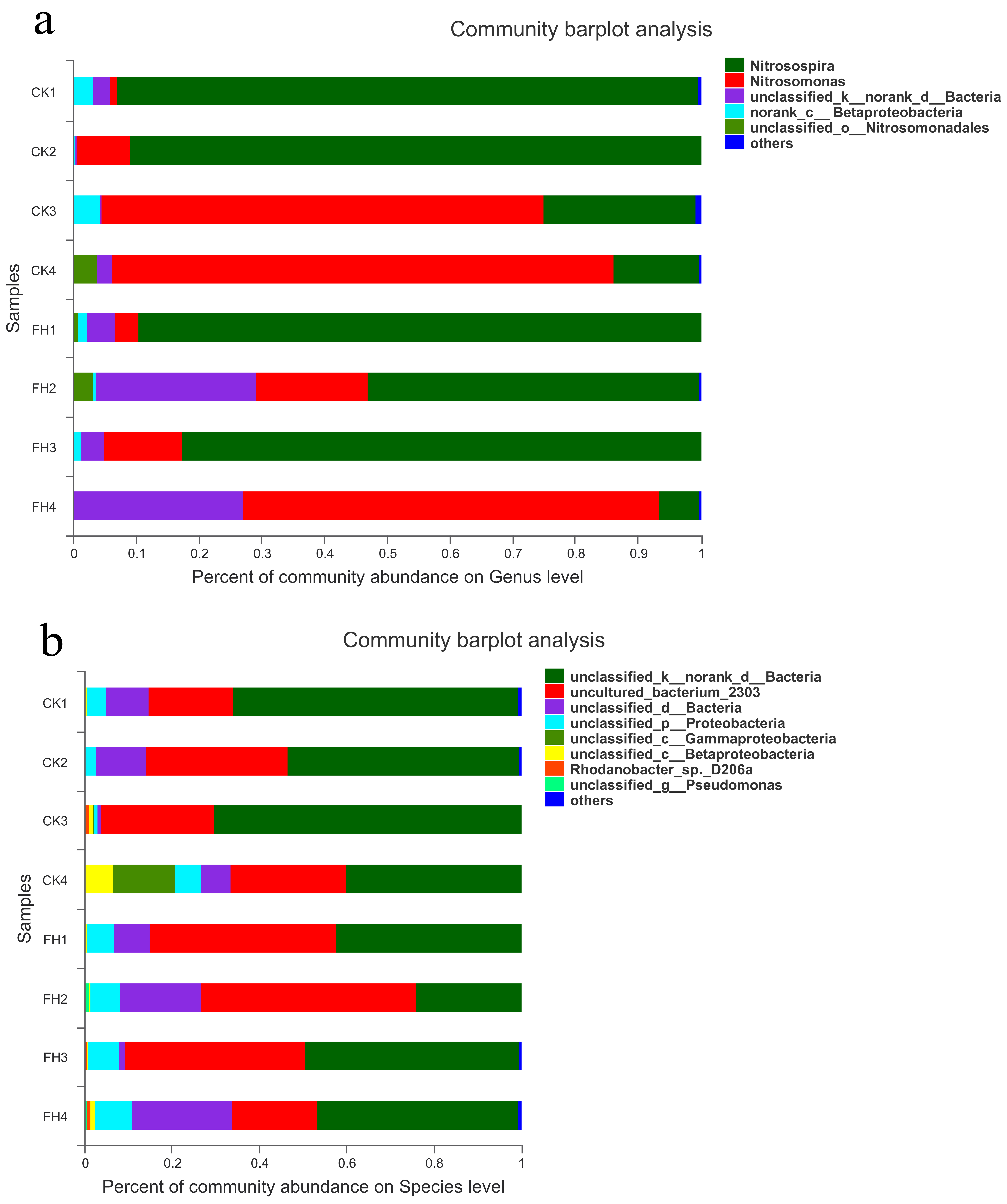
a
Community barplot analysis
Nitrosospira
Nitrosomonas
unclassified_k__norank_d__Bacteria
norank_c__Betaproteobacteria
unclassified_o__Nitrosomonadales
others
CK1
CK2
CK3
CK4
FH1
FH2
FH3
FH4
Samples
0
0.1
0.2
0.3
0.4
0.5
0.6
0.7
0.8
0.9
1
Percent of community abundance on Genus level
b
Community barplot analysis
unclassified_k__norank_d__Bacteria
uncultured_bacterium_2303
unclassified_d__Bacteria
unclassified_p__Proteobacteria
unclassified_c__Gammaproteobacteria
unclassified_c__Betaproteobacteria
Rhodanobacter_sp._D206a
unclassified_g__Pseudomonas
others
CK1
CK2
CK3
CK4
FH1
FH2
FH3
FH4
Samples
0
0.2
0.4
0.6
0.8
1
Percent of community abundance on Species level
